# Use of Plant Extracts as an Effective Manner to Control* Clostridium perfringens* Induced Necrotic Enteritis in Poultry

**DOI:** 10.1155/2016/3278359

**Published:** 2016-09-25

**Authors:** J. M. Diaz Carrasco, L. M. Redondo, E. A. Redondo, J. E. Dominguez, A. P. Chacana, M. E. Fernandez Miyakawa

**Affiliations:** ^1^Instituto de Patobiología, Centro Nacional de Investigaciones Agropecuarias, Instituto Nacional de Tecnología Agropecuaria, Calle Las Cabañas y Los Reseros s/n, Casilla de Correo 25, 1712 Castelar, Buenos Aires, Argentina; ^2^Consejo Nacional de Investigaciones Científicas y Técnicas, Rivadavia 1917, 1033 Ciudad Autónoma de Buenos Aires, Argentina

## Abstract

Necrotic enteritis (NE) is an important concern in poultry industry since it causes economic losses, increased mortality, reduction of bird welfare, and contamination of chicken products for human consumption. For decades, the use of in-feed antimicrobial growth promoters (AGPs) has been the main strategy to control intestinal pathogens including* Clostridium perfringens* (CP), the causative agent of NE. However, the use of AGPs in animal diet has been linked to the emergence and transmission of antimicrobial resistance through food-borne microorganisms, which has led to the ban of AGPs in many countries. This scenario has challenged the poultry industry to search for safer alternative products in order to prevent NE. In this context, the utilization of natural plant extracts with antimicrobial properties appears as a promising and feasible tool to control NE in chicken. In this paper, we review the scientific studies analyzing the potential of plant extracts as alternative feed additives to reduce NE in poultry, with focus on two types of plant products that arise as promising candidates: tannins and essential oils. Some of these products showed antimicrobial activity against CP and coccidia* in vitro* and* in vivo* and are able to increase productive performance, emulating the bioactive properties of AGPs.

## 1. Necrotic Enteritis in Chickens and* Clostridium perfringens*

Necrotic enteritis (NE) is a worldwide extended disease caused by* Clostridium perfringens* (CP). The disease was first reported in 1961 and from that moment onwards many outbreaks have been documented in all countries where intensive poultry breeding is carried out [1–3]. NE has different presentations: sudden, clinical, and subclinical; among them, subclinical NE is one of the main causes of economic loss for the poultry industry. The estimated prevention cost of NE is U$S 0.05 per chicken with a total global loss of nearly U$S 2 billion per annum [4]. CP is a ubiquitous Gram-positive, spore forming, toxigenic, anaerobic bacterium, generally classified according to the production of five major toxins [5]. In poultry industry, CP type A is the most significant, since it is capable of producing many toxins responsible for the disease [6].

CP can be found in the environment in soil, feces, feed, and poultry litter and in the intestines of animals as part of the normal gut microbiota [7]; thus, the presence of CP by itself does not necessarily imply the occurrence of the disease. NE reports showed that the disease is mostly found in 2- to 5-week-old chickens and the incidence of the disease can be low as well as high, as most CP strains are relatively innocuous. Clinical presentation of NE in outbreaks depends on a complex interaction of the microorganism with other predisposing factors such as diet, the presence of other microorganisms, and the immunological status of the birds [2, 4, 8, 9]. The ingredients included in diet, and even changes in it, may affect both physical and chemical properties of intestinal contents. Presence of* Eimeria *spp. and viral infections are important NE-predisposing factors as they lead to the destruction of enterocytes and increase the mucosal secretion. Stress, immunosuppression, or medical treatment can also induce changes in the composition of the microbiota. All of these factors contribute to facilitating the mucosal colonization for pathogenic CP strains, which are able to degrade the mucus and colonize the gut. When this happens, the bacteria begin to synthesize enzymes, acting in the basement membrane and lateral part of the enterocytes, spread through the lamina propria, and induce damage to endothelial cells [6, 10–13].

Clinical signs of NE include decreased appetite, diarrhea, weight loss, and several nonspecific signs that can be found even without any gut lesion [1, 2]. Gross lesions are diverse, usually affecting the small intestine and liver; jejunum and ileum are the most affected portions of the gut. Intestines are visualized with gas as well as bleeding and blood clots can be found in their contents. The mucosa can be either thickened by edema or thinned by epithelial erosion [1, 2, 6, 8] and sometimes a yellow or green pseudomembrane adhered to mucosa can be found. It is likely to find in the same animal both changes in different parts of the intestine [1–3, 6]. In the liver, necrotic foci and cholecystitis can appear dispersed throughout the parenchyma. These injuries are commonly associated with a subclinical presentation of the disease [2, 14]. Microscopic lesions comprise shortening villous, epithelium detachment in the apical portion, and also intense mucosal necrosis extending to the crypts or submucosa. Bacilli can sometimes be seen in the mucosa or in lamina propria. The inflammatory cell infiltration in lamina propria is a mixed type and more evident in some cases than others [1–3, 8].

The treatment of NE outbreaks are based on antimicrobial therapy with the aim of diminishing economic losses. Bacitracin, lincomycin, virginiamycin, penicillin, and tylosin have been the antibiotics of choice worldwide. However, the most important losses are associated with subclinical presentation of NE, which has been controlled by the use of subtherapeutic doses of antimicrobials in feed [15]. As it happens with several microorganisms, CP susceptibility to antibiotics has declined over the years.

## 2. Antimicrobial Control of NE and Alternatives

Control of NE and predisposing factors in poultry often becomes a really complex labor. For many years, antimicrobial therapy was the first, and most of the times the only, strategy to control CP-induced NE. Therapeutic antimicrobials administered at high doses over a short period of time are generally used to control acute outbreaks [16]. To control subclinical NE presentations, antimicrobial growth promoters (AGPs) are generally used. Although these compounds were first included into feed to improve growth rate and feed conversion efficiency in poultry [17], they are now used mostly to control CP and other Gram-positive pathogens [18]. Bacitracin (a polypeptide antimicrobial) and virginiamycin (a streptogramin) are nowadays two commonly used AGPs in poultry production to improve feed conversion ratios, body weight gain, and well-being of animals [16]. Despite the longtime use of AGPs, mechanisms involved in the improvement caused by the administration of subtherapeutic doses of the antimicrobials in broilers flocks are far from being fully understood. Proposed potential mechanisms include regulation of digestive functions and gut immunological responses [19]. The most accepted mechanism is that AGPs modulate gut microbiota, which plays a critical role in maintaining the host health [20].

The use of AGPs at subinhibitory doses for long periods of time is particularly favorable to select antimicrobial-resistant microorganisms. In countries where AGPs are still used, reduced susceptibility of poultry CP strains was reported [21]. Continuous administration of AGPs may lead to changes in the bacterial environment by eliminating susceptible strains and allowing antimicrobial-resistant bacteria (i.e., those with lower susceptibility to AGPs) to survive and predominate [22]. Furthermore, continuous administration of APGs in the feed may cause cross-resistance to therapeutic antimicrobials [21]. Antimicrobial resistance together with a gradual decrease in sensitivity to anticoccidials by some strains of* Eimeria *spp. (an important predisposing factor to NE) has exacerbated the presence of such CP strains.

Establishment of resistant and pathogenic CP strains in poultry farms also may lead to the transfer of resistant bacteria and their resistant factors from animals to humans. Studies considering* Campylobacter *spp.,* E. coli,* and* Enterococcus *spp. suggest that the use of nontherapeutic antimicrobial is linked to the propagation of multidrug resistance, including resistance against drugs that were never used in the farm [23].

The impact of AGPs on the appearance and transmission of antimicrobial-resistant bacteria has been the aim of several investigations and has led to their ban in the European Union in 2006 [24]. After these measures were taken in Europe [25], the consequence was the increase in NE incidence together with an increase in the use of therapeutic antimicrobials to control diseases [26–28]. The European experience and recent moves toward reduction or termination of AGPs in North America [25] have pressured the poultry industry to search for suitable alternatives in order to control NE outbreaks, reduce productive consequences of subclinical presentation under conditions of average management of the farms [26], and diminish resistance to antimicrobials. Natural substances with antimicrobial properties can be an essential part of this control strategy.

In this context, an increasing number of antimicrobial-free tools and strategies have been developed for prevention and control of CP-induced NE in poultry [27, 29]. Any alternative to AGPs is expected to be safe to the public health, cost-effective, and friendly to the environment together with antimicrobial activity to be considered as a viable option [30]. Proposed alternatives include vaccines, immunomodulatory agents, bacteriophages and their lysins, antimicrobial peptides, pro-, pre-, and synbiotics, plant extracts, inhibitors for bacterial quorum sensing, biofilm, and virulence and feed enzymes [27, 31]. Vaccination against the pathogen and the use of probiotic and prebiotic products have been suggested but at the present time are not yet available for practical use in the farms. One of the most promising alternatives to AGPs is the use of plant extracts added to the diet to improve nutrition and health in farm animals and to control enteric clostridial diseases; these additives have been used for many years in poultry and their efficiency has been demonstrated [21, 32].

## 3. Plant Extracts

Plant materials are used widely in traditional systems of medicine [55]. Plant extracts, also known as phytobiotics, have been exploited in animal nutrition, particularly for their antimicrobial, anti-inflammatory, antioxidant, and antiparasitic activities [56–58].

Many plants have beneficial multifunctional properties derived from their specific bioactive components. Biologically active components of plants are mostly secondary metabolites, such as terpenoids, phenolics, glycosides, and alkaloids, present as alcohols, aldehydes, ketones, esters, ethers, and lactones [17]. These secondary metabolites may have a protective function in vegetal tissues. Final effect on animals will depend on both the combination and concentration of these bioactive molecules and minor changes in these aspects will explain why some of these compounds can have either beneficial or detrimental effects in animals [59–61].

Plant extracts are generally considered safe and effective against certain bacteria. They are extensively used in feed as growth promoters and health protectants [62, 63], particularly in Asian, African, and South American countries, and in recent years are gradually being used in developed countries. Effects of phytogenic feed additives on the production performance of poultry are also reported [57]. It is considered that plant extracts possess antibacterial activities when their minimum inhibitory concentrations range from 100 to 1000 *μ*g/mL according to* in vitro* bacterial susceptibility tests [64].

In the last years, several studies reported that the use of raw plant extracts and derived phytogenic compounds as poultry feed supplements [65] may have a positive effect on birds health and productivity. NE gross lesions and intestinal CP burden is a parameter commonly used to evaluate the effects of including plant extract in chicken diets [35, 47]. Different plant extracts may have direct inhibitory effect either on CP vegetative cells or in some of the toxins involved in NE pathogenesis [32].

Useful antimicrobial phytochemicals can be divided into several categories, such as polyphenols/tannins, essential oils (EOs), alkaloids, and lectins/polypeptides [66]. Phytochemicals exert their antimicrobial activity through different mechanisms. For example, (1) tannins act by iron deprivation and interactions with vital proteins such as enzymes [67]; (2) cryptolepine, the main indoloquinoline alkaloid, is a DNA intercalator and inhibitor of topoisomerase [68]; and (3) saponins form complexes with sterols from the membrane of microorganisms causing damage and consequent collapse of the cells [69]. EOs have long been recognized for their antimicrobial properties [70], but their precise antimicrobial mechanism is poorly understood. In fact, the antimicrobial activities of many plant extracts have not been clearly elucidated yet [71].* In vivo* observations support the assumption that general antimicrobial potential of phytogenic feed additives is due to a substantial reduction of intestinal pathogen pressure [66].

In the global context to reduce or avoid the use of antimicrobials in animal production, not only biological activity of alternatives to AGPs but also the suitability of the active principles to be produced and applied at the industrial level should be considered. In the last years, two types of plant-derived extracts emerged as promising candidates to be used in poultry industry to control NE: tannins and essential oils.

## 4. Tannins

Tannins are polyphenolic secondary metabolites found in almost all the parts of the plants and therefore present in most animal diets. Tannins are generally classified into two groups based on their chemical structure: hydrolyzable tannins (HT) which are present in plants as gallotannins or ellagitannins [72] and condensed tannins (CT), the most common type of tannins found in forage, which are polymers of flavonol units [73]. However, tannins have an enormous structural diversity, with molar masses ranging from 300 to 20,000 Da [74]. Multiple biological properties including anticancer [75, 76] and antimicrobial [67, 77, 78] activities have been attributed to tannins [79], mainly due to their ion-complexation, protein-binding, and antioxidant capabilities [80–82].

Scientific evidence suggests significant potential for the use of tannins to enhance nutrition and animal health in both cattle and poultry [30, 62, 65, 83]. Although tannins have been generally considered as antinutritional factors for monogastric animals [59, 84, 85], it is now known that their beneficial or detrimental properties depend on both tannin nature (i.e., plant source, chemical structure, and astringency) and animal factors (i.e., animal species, physiological state, and diet composition) [39, 57, 61, 66, 86, 87] as well as administration factors such as dosage and formulation. The antinutritional effects attributed to tannins are mostly based on assays performed with elevated concentrations of CT or plant parts with elevated tannin content, as may be the case of tannic acid in sorghum.

In recent years, many reports showed that moderate tannin concentrations from diverse vegetal sources can improve not only nutrition but also health status in monogastric farm animals, including poultry. Furthermore, inclusion of polyphenol-rich plant extracts has been found to improve weight gain/feed ratio in growing pigs [88]. In poultry, Schiavone et al. (2008) [34] showed that the use of a chestnut extract has a positive influence on growth performance if included in the diet up to 2 g/kg of dry matter and also a significant decrease in total nitrogen in the litter was observed. This supports the observation that administration of chestnut tannins often results in firmer droppings, which positively affects the litter status thus improving the overall health status and welfare of chickens in intensive production systems. Similarly, other authors have observed that the inclusion of phenolic compounds in diet enhanced growth performance, decreased lipid oxidation, decreased cholesterol value, and increased beneficial fatty acids content in broiler chickens [89]. However, other tannin formulations are unable to enhance growth performance but produce different beneficial effects in productive aspects of chicken physiology, including delay of meat lipid oxidation [37, 41, 90–92], increase of protein digestibility and feed conversion [38, 42], enhancement of gut health and microbiota biodiversity [40, 93, 94], and higher capacity to overcome deleterious effects of persistent heat stress [95, 96].

Numerous* in vitro* and* in vivo* studies have verified the activity of tannins against several types of intestinal pathogens including helminthes [97–100], coccidia [33, 36, 101], viruses [45, 77], and bacteria [102–104] with particular interest in* Salmonella* Typhimurium [44, 105, 106],* Campylobacter jejuni* [43], and CP [32, 35], which are major disease-causing or food-borne bacteria in poultry [107].

Incidence of CP-associated NE in poultry has considerably increased in countries that stopped the use of AGP [26, 108]. Elizondo et al. (2010) [32] showed that two of the most common sources of tannins, chestnut (*Castanea sativa*, HT) and quebracho (*Schinopsis lorentzii*, CT), extracts have* in vitro* antibacterial and antitoxin activities against CP and its toxins. Similarly, other authors have observed* in vitro* antimicrobial activity against CP using tannins derived from chestnut and grape products [40, 109].

These findings are consistent with recent* in vivo* studies that tested the effect of tannins added to diet of chickens on* Eimeria *spp. and CP. Tosi et al. (2013) [35] found that the addition of a chestnut tannin extract significantly reduces the counts of CP and macroscopic gut lesions in broiler chickens challenged with coccidia and CP. Subsequent results confirm the effects of chestnut and quebracho tannins in a broiler NE model reducing the incidence and severity of gross lesions and improving the productive performance of the chicken [110]. Although chestnut tannins show strong bactericidal activity against CP, most ingested HT are degraded in the intestinal tract and do not remain in the feces. In contrast, quebracho-derived CT have lower antibacterial activity but most of the administered tannins remain in the feces and therefore in the litter. Combination of CT and HT may be used to readily diminish the intestinal CP load and also to avoid the reinfection by controlling the environmental contamination (i.e., feces and bedding). In agreement with this, Cejas et al. (2011) [36] found that quebracho tannins also decreased oocyst excretion in* Eimeria *spp. challenged broiler chicks. Consistent results were also obtained with other tannin-rich plant extracts. McDougald et al. (2008) [33] showed that inclusion of muscadine pomace in the diet significantly reduced intestinal lesion scores and mortality rates using a similar NE model of broilers challenged with* Eimeria *spp. and CP. Dietary supplementation of chicken diet with a polyphenol extract of* Curcuma longa* enhanced coccidiosis resistance as demonstrated by increased body weight gains, reduced fecal oocyst shedding, and decreased gut lesions, and it was also shown to attenuate coccidia-induced inflammation-mediated gut damage [101].* Artemisia annua* leaves, which contain both EOs and tannins [111], showed antimicrobial activity against CP proliferation* in vitro* and were able to reduce intestinal load and severity of NE-related small intestinal lesions* in vivo* [47].

A recent work reported that chestnut extracts improve lactobacilli tolerance to gastric transit and tolerance to low pH values and bile juice salts, indicating that tannins may also be used in combination with probiotics for synergist enhancement of gut health [112]. An additional benefit of the use of tannins as alternative AGPs in poultry is the difficulty of CP to multiply and develop resistance in the presence of such diverse range of molecules these plant compounds contain [21].

Although tannins can have beneficial effects on poultry performance and gut health, still little is known about the mechanisms involved in their final* in vivo* antimicrobial and growth promoter effects. Some authors suggest that low concentration of tannins can improve palatability of feed thus increasing performance of monogastric animals by stimulating feed intake [66]. Nevertheless, antimicrobial activity has been linked to their biochemical properties including metabolism inhibition by enzyme complexation and iron deprivation [67, 80, 113, 114]. Iron is essential for most pathogenic bacteria and tannic acid has been shown to function like a siderophore that chelates iron from the medium, making it unavailable for some microorganisms but without affecting lactic acid bacteria [102]. Regarding the growth promotion effect, some of the explained modes of action for antimicrobials may help to define tannin mechanisms. How antimicrobials increase performance is not clear, but possible mechanisms include reduction in total bacterial load, suppression of pathogens, thinning of the mucosal layer, and direct modulation of the immune system [115]. In general terms, like AGPs, tannins may be involved in the modulation of gut microbiota and its highly complex interactions. As reported by several authors, Gram-positive bacteria seem to be more sensitive to tannin-rich plant extracts [104, 116]. Regardless of the mode of action, chemical characteristics of the tannins are highly variable and different types of tannins can be found in a single plant extract. The origin of the plant extract added to the feed will be determinant in the final impact on microbiota and consequently in growth performance.  Table 1 summarizes the effects of different tannin-rich plant extracts on performance and health of poultry* in vivo* and their antimicrobial activities* in vitro*.

The use of tannins appears as an attractive alternative to control NE since these natural products do not leave residues in poultry-derived products and given the complexity of their structures and bioactive principles it is more difficult for tannins to induce selection of resistant microorganisms in comparison with AGPs. Among the wide range of tannin-rich plant extracts with beneficial effects in poultry nutrition and health, chestnut and quebracho tannins are probably the most readily available commercial products that are being used and there are a significant number of publications that demonstrate their properties. Further research needs to be done in order to elucidate the mechanisms associated with antimicrobial activity of tannins as well as their impact on the development of a healthy gut microbiota in poultry.

## 5. Essential Oils

Essential oils (EOs) are considered to be secondary metabolites in plants which are organic compounds that are not directly involved in the normal growth, development, or reproduction of the plant [117]. These compounds are assumed to be involved in plant defense and most of them may possess antimicrobial properties [117, 118].

The composition and the percentage of different components of EOs vary amongst species and parts of the plants; most of these components are chemically derived from terpenes and their oxygenated derivatives, terpenoids, which are aromatic and aliphatic acid esters and phenolic compounds. EOs can be extracted from plant tissues by extraction or fermentation, but steam distillation is the most commonly used method in industry. EOs have been historically included in the formulation of perfumes and cosmetic products as well as herbs and spices for foods. These oily components are generally recognized as safe (GRAS) by the Food and Drug Administration (FDA) of the United States and have been used as artificial flavorings and preservatives [119]. Also, herbs and spices and their EOs have been used as pharmaceuticals in alternative or complementary medicine for many years [120].

Recently, there was a renewed interest on the antimicrobial activity of EOs since many reports demonstrated the potential to control bacterial pathogens [121–123]. The first scientific test of their bactericidal properties had been carried out by de la Croix in 1881 [123]. In more recent years, many EOs or their components have been shown to possess broad-range antibacterial properties [124, 125].

Antimicrobial activities of EOs are related to chemical characteristics such as their hydrophobicity which enables them to interact with the lipids of the bacterial cell membrane thus disturbing bacteria metabolism and cell wall and membrane permeability, leading to extensive leakage of critical molecules and ions from bacterial cells. Phenolic groups present in EOs molecules target bacterial cell membrane by changing its structure and function [126]; microscopy studies demonstrate that low concentrations of some oils may generate holes on the cell wall of sensitive bacteria including CP, being vegetative forms particularly lysed [127].

Evidence about inhibitory spectrum of EOs is contradictory. Some studies concluded that Gram-positive bacteria are more resistant than Gram-negative bacteria [128]. However, most works reported that Gram-positive bacteria are more susceptible to EOs than Gram-negative bacteria [123, 129]. The weaker antimicrobial activity against Gram-negative can be explained considering the structure of their cellular walls, mainly with regard to the presence of lipoproteins and lipopolysaccharides in the external membrane that form a barrier to hydrophobic compounds [129, 130].

Unlike common antibiotics that are often composed of only a single molecular entity, EOs are multicomponent substances and the antibacterial efficacy is related to the overall composition and relative concentrations of active components. For example, thymol and carvacrol, two common terpenoids present in many EOs, have similar antimicrobial properties but act differently against Gram-positive or Gram-negative bacteria based on the locations of one or more functional groups in these two molecules [30, 131]. The mechanism underlying antibacterial activity against CP and other Gram-positive bacterial pathogens is unclear at present and therefore further studies are needed.

The use of EOs to control the proliferation of CP and reduce NE impact on poultry production has been explored [48, 49, 53, 54]. There are numerous reports about the antibacterial effects of* Origanum vulgare*,* Piper nigrum*,* Syzygium aromaticum*, and* Thymus vulgaris*, and their components, thymol, carvacrol, and eugenol, against* Clostridium* species [132, 133] including CP [46, 134]. EOs effects on CP-induced NE may be related to a direct antimicrobial effect on bacterial cells and an indirect effect by modulating gut microbiota and digestive functions.* In vitro* CP inhibition was described for many plant extracts and their EOs [127]. Antimicrobial activity was found in 50% of the tested plant species. Great differences in the inhibitory effect and potency are found among scientific studies on the activity of EOs that can be partially explained by the variety of protocols used to obtain EOs solution and to measure antimicrobial activity [127, 135]. For example, one report [135] used disc diffusion methods and reports high antimicrobial activity (inhibition > 95%) against CP for thyme (*T. vulgaris*) and oregano (*O. vulgare*), while Si et al. (2009) [127] used broth microdilution methods and reported similar results for thyme but low antimicrobial activity (inhibition between 50 and 80%) for oregano. Since antimicrobial activity of EOs is related to the combined effects of several molecules, most reported results choose one of the main components as indicator of biological activity. Carvacrol and thymol are two of the most common single molecules used to determine spices/EOs antimicrobial activity [50, 123], and differences in presence and concentration of these molecules will contribute to explain the contradiction of published results. The aforementioned molecules are main components of several EOs with antimicrobial activity such as oregano, rosemary, and thyme oils [123, 136].

Differences in activity may also be related to vegetal growth conditions and storage conditions after harvest [137]. These authors compared several commercial stocks of spices Angelica (*Angelica archangelica*) and Japanese mint (*Mentha arvensis* var.* piperascens*) and found clear differences in antimicrobial activity [137]. Some works also reported variations in thymol and carvacrol concentrations within thyme and oregano [137]. Moreover, while some works mention that carvacrol is the main active molecule in thyme, Nevas et al. (2004) [137] described inhibitory effect against CP in Finnish thyme extract without detection of carvacrol.

In poultry, many works report that the inclusion of blends of EOs as dietary supplements has improved productive performance [52, 138] including weight gain and body mass; however, none of these works reported changes in intestinal microbiota, apparent metabolizable energy, or the calculated coefficients of digestibility. According to Jamroz et al. (2003) [139], the inclusion of blended supplements containing carvacrol, capsaicin, and cinnamaldehyde has improved body weight and feed conversion rate in broilers even to a greater extent than avilamycin in 21-day-old chickens.

The inclusion of EOs supplementation in poultry feed alleviated intestinal gross lesions compatible with NE in a dose-dependent manner on days 21 and 28 [50, 140]. Reduction of CP-induced intestinal damage can be achieved after reducing the intestinal burden of the microorganism. Si et al. (2009) [127] reported reductions of 2 or 3 log units of CP counts in chicken ileal content by carvacrol or citronellol; these results agreed with previous* in vivo* studies which showed that EOs containing thymol and/or carvacrol were able to decrease CP counts in both small and large intestines [51].

One important criterion that may be considered to select good candidates for the substitution of AGPs to control CP-induced NE and other poultry bacterial pathogens is their stability at low pH, as all compounds need to pass through the stomach with a pH as low as 2. Some EOs like carvacrol, chamomile roman oil, or citronellal resist acid and retained their inhibitory activity toward CP after the* in vitro* preacidic treatment [127]. These results suggest that some EOs can be added to feed and have intact effect against CP vegetative cells located in the intestinal lumen.* In vivo* trials support this since they showed that birds fed with EOs supplemented had lower concentrations of CP in jejunum, cecum, cloaca, and feces on day 14, in jejunum, cecum, and feces on day 21, and feces on day 30. Chickens fed with EOs showed significantly lower CP counts in all portions of the intestine and in the feces, while the proportion of CP positive birds was also reduced [46]. Unlike tannins [32], no antitoxic activity against CP toxins was demonstrated for EOs.

Together with direct antimicrobial effects of EOs against CP, changes in intestinal microbiota also might be related to alleviation of development of NE intestinal lesions. Several studies have reported that changes in intestinal microbiota induced by essential oil dietary supplementation are to the same extent as avilamycin [139]. Once again, evidence is contradictory and needs to consider variations on EOs origins as well as feed supplement presentation. Cross et al. (2007) [52] reported that the inclusion of rosemary (*R. officinalis*), yarrow (*A. millefolium* var.* alba*), and thyme (*T. vulgaris*) in poultry diets reduced CP counts in cecum and increased coliforms counts in the same intestinal portion in chickens given any of the mentioned herbal treatments. Other works mention that blends of EOs reduce the growth of* E. coli* and CP in broilers [141, 142]. EOs exhibited a minor or no inhibition on* Lactobacillus *spp. [52] and some works report an increased number of lactobacilli counts [142]. Thus, EOs may act in a different way compared to AGPs, which tend to depress bacterial numbers in all the species. While some works report higher susceptibility to EOs in Gram-positive bacteria, other studies demonstrated the selectivity of EOs against CP over lactobacilli, both groups of Gram-positive bacteria. Undoubtedly, further studies are required to understand the mechanism underlying the group selectivity.

To control CP-induced NE and other infectious diseases, it is important to reduce intestinal and environmental bacteria burden. Some EOs formulations also reduce bacterial populations when applied directly on the soil and can be used to reduce potential contamination of fresh organic products, including poultry feed. Previous works with different bacterial pathogens on food products intended for human alimentation, including products of plant or animal origin, suggest a promising scene [47, 143, 144]. In the actual poultry productive context where synthetic antimicrobials are limited or banned, EOs could play an important role in the innovation of preventive or therapeutic strategies. It is likely that it will be more difficult for bacteria to develop resistance to the multicomponent EOs than to common antibiotics that are often composed of only a single molecular entity. Previous works with tannins [21], another multicomponent natural antimicrobial substance, may reinforce this idea. Nevertheless, the lack of studies to determine the safety and toxicity evaluation of potential changes in flavor, odor, and other organoleptic characteristics of poultry-derived food products may limit the use of EOs in poultry. Available information regarding safety in relation to oral administration of EOs in human and poultry is scanty, so determinations upon the potential toxicity of EOs administered by this route are required. The ways in which EOs are applied and the concentrations at which they are used are important factors related to their effectiveness. Inhibition studies showed that some pathogenic bacteria can be inhibited by direct application of EOs components without affecting the flavor of the food products [145].  Table 2 summarizes the available EOs additives for NE prevention in poultry as well as their performance and intestinal and antimicrobial effects.

## 6. Conclusions and Perspectives

The European ban of AGPs in poultry products and recent restrictions on the use of these compounds in other countries, including Australia and USA, present several challenges to the poultry industry. Reports from the EU have shown that the key problem of in-feed antibiotic withdrawal from poultry diets is the control of NE. Therefore, the cost-benefit in replacing AGPs with natural alternatives is critical for ensuring the long-term sustainable poultry production. Plant extracts have a large variety of bioactive ingredients and thus represent one of the most promising alternatives to replace AGPs, particularly tannins and essential oils. However, their application in poultry production has been largely avoided due to inconsistent evaluation of their efficacy and lack of full understanding of the modes of action behind them. In order to support the use of natural plant products to maintain the productivity rates achieved by AGPs and become acceptable by the mainstream poultry industry market, different research groups provided solid scientific evidence addressing the issue of inconsistency across many studies in literature. In this sense, the development and utilization of a standardized methodology for production of phytobased feed additives and evaluation of their biological activity is urgently needed in order to support the use of different additives. Furthermore, a better understanding on the impact of phytogenic compounds on gut microbiota, physiology, and immunology will allow a better use of these products for economically effective and sustainable poultry production.

Besides plant extracts, there are other suitable strategies to control NE in poultry in order to fill the gap left by the ban of AGPs, including competitive exclusion products, probiotics, prebiotics, organic acids, enzymes, hen egg antibodies, bacteriophages, and vaccination. However, to date, no single preventive therapy that can effectively substitute AGPs and control NE has been found. Therefore, the combination of different in-feed additives and limiting exposure to CP and other NE-predisposing microorganisms through biosecurity and vaccination might be a tool to reduce the incidence of NE and improve gut health in the absence of AGPs. Effective nonantibiotic prevention of CP-associated health and performance problems will only be achieved by means of multidisciplinary research efforts, covering both* in vitro* molecular functionality approaches together with* in vivo* feeding experiments. Plant extracts exert specific effects on gut microbiota which influence both the emergence of intestinal pathogens and growth performance of chickens. It has been shown that tannins and essential oils possess activities in the digestive tract that cover many of the requirements to control NE. The ability of some tannins to remain active even in poultry bedding after fecal excretion appears as an interesting feature to control CP reinfection. Moreover, it has been proved that resistance of CP against tannins is not easily generated, allowing a continuous use of these compounds over time. Therefore, these products may play a key role as a viable, cost-efficient, and safe alternative to AGPs that could be used to enhance chicken performance and health as well.

## Figures and Tables

**Table 1:** *In vivo* and *in vitro* effects of tannin-rich plant extracts on performance and health of poultry.

Tannin source and type	Major findings		Doses (g/kg)	References
*In vivo effect of tannin-rich plant extracts on performance and health of poultry*				
	Performance effects	Health effects		

Muscadine pomace (HT and flavonoid; *Vitis rotundifolia*)	Birds given 50 g/kg had poorer average live weight. Extracts at 5 and 20 g/kg improved BWG after challenge with CP in a NE model.	Treated birds showed increased resistance to coccidia infection and lower lesion scores after challenge with coccidia (*Eimeria *spp.). Extracts at 5 and 20 g/kg reduced mortality and lesion scores using an established model of NE.	5, 20, and 50	[33]

Chestnut (HT; *Castanea sativa*)	Chestnut extract did not influence feed digestibility, carcass quality, or N balance and showed a positive influence on growth performance up to 2 g/kg.	Carcass analysis showed no gross lesions in organs and no significant differences in thigh and breast composition among groups.	1.5, 2, and 2.5	[34]

Chestnut (HT)	—	Chestnut extract reduced the counts of CP and macroscopic gut lesions in a NE model challenged with coccidia and CP. Results were more pronounced at higher tannins doses.	1.5, 3, 5, and 12	[35]

Quebracho (CT; *Schinopsis lorentzii*)	Quebracho supplementation significantly increased BWG and intestinal V : C ratio.	Birds challenged with coccidia (*Eimeria *spp.) and treated with quebracho extract showed decreased oocyst excretion. No differences in mortality or intestinal lesion were observed.	100	[36]

Grape pomace (CT)	No negative effects on growth performance, digestive organ sizes, or protein digestibility were detected at levels up to 60 g/kg to 42 d of age.	Antioxidant activities in diet, excreta, ileal content, and breast muscle were increased by grape pomace concentrate.	15, 30, and 60	[37]

Grapeseed (CT; *Vitis vinifera*)	Inclusion of grapeseed extract up to 3.6 g/kg did not affect growth performance and increased protein and polyphenol digestibility.	Grapeseed extract caused a significant increase of antioxidant activity in diet and excreta.	0.6, 1.8, and 3.6	[38]

Grapeseed, mimosa (CT), and cranberry (CT + HT)	Mimosa extract reduced BWG and FCR while cranberry reduced feed digestibility.	Mimosa and cranberry supplements decreased minor VFA concentration, which is probably associated with inhibition of microbiota activity.	373, 258, and 24, respectively	[39]

Grape pomace and grapeseed (CT)	Grapeseed diet showed decreased BWG. Both extracts reduced intestinal crypt depth and increased muscular thickness.	Both extracts decreased the counts of *Clostridium* in ileum but these were increased in cecum. Only grapeseed extract showed antimicrobial activity against CP.	60 and 7.2, respectively	[40]

Grape pomace (CT)	No differences in BWG or FCR were observed. Birds fed with grape pomace diets had a higher content of meat polyunsaturated fatty acids.	Grape pomace added up to 100 g/kg prevented meat lipid oxidation similar to vitamin E.	50 and 100	[41]

Quercetin (flavonoid)	FCR decreased as quercetin level increased. Laying rate was maximized by supplementation of quercetin at 0.2 g/kg. No significant effect on egg quality was observed.	Microbial population of aerobes and coliforms decreased at higher levels of quercetin, while bifidobacteria increased. Antioxidant activity in liver was increased by quercetin.	0.2 to 0.6	[42]

*Tannin source and type*	*Major findings*		*Doses (mg/mL)*	*References*

*In vitro effect of tannin-rich plant extracts on CP and other poultry pathogens*				

Chestnut (HT) and quebracho (CT)	CP types A, B, C, D, and E growth was inhibited in a dose-dependent manner in the presence of all tannins extracts. Quebracho tannins showed partial bactericidal activity, whereas chestnut tannin activity was stronger. Both tannins reduced alpha toxin lecithinase activity and epsilon toxin cytotoxicity.		0.03 to 8	[32]

Blackberry, cranberry (CT + HT), chestnut (HT), mimosa, quebracho, and sorghum (CT)	Tannins exhibited a variable degree of inhibition of *Campylobacter jejuni* depending on source and medium composition. Inhibitory activity of CT extracts but not HT was mitigated by casamino acid supplementation, likely because the added amino acids saturated the binding potential of CT.		100	[43]

Chestnut, tara, sumach (HT), quebracho, *Calliandra* (CT), green tea, and acacia (flavonoid)	Despite their structural diversity, all tested tannins showed antimicrobial effects against *Salmonella* Typhimurium either in liquid culture or in the diffusion assay. Tara extracts effectively reduced the counts of *Salmonella* Typhimurium at low doses (1 mg/mL).		1 to 6	[44]

Chestnut (HT) and quebracho (CT)	All compounds showed extracellular antiviral effect against both avian reovirus and avian metapneumovirus at concentrations ranging from 25 to 66 *μ*g/mL. Quebracho extract also had intracellular activity.		0.01 to 0.15	[45]

CP: *C. perfringens*; BWG: body weight gain; HT: hydrolyzable tannins; CT: condensed tannins; FI: feed intake; FCR: food conversion rate; NE: necrotic enteritis.

**Table 2:** EOs additives for NE prevention in poultry.

Feed additive	Inclusion rate	Performance effects	Intestinal effects	Antimicrobial effects	Reference
EOs from *Thymus vulgaris* (30% thymol)	100 ppm	—	—	Decreased CP counts in the gut and feces.	[46]

EOs mix from *Thymus vulgaris* and *Origanum vulgare *(15% thymol and 15% carvacrol)	100 ppm	—	—	Decreased CP counts in the gut and feces.	[46]

*Artemisia annua* dry leaves	10 g/kg	Decreased BWG. Decreased FI. Improved FCR.	Reduced NE-related gross intestinal lesion score.	*In vitro* antibacterial activity against CP. Decreased CP counts in ileum and ceca.	[47]

*Artemisia annua* n-hexane extract	250 mg/kg	Decreased BWG. Decreased FI. Improved FCR.	Reduced NE-related gross intestinal lesion score.	*In vitro* antibacterial activity against CP. Decreased CP counts in ileum and ceca.	[47]

*Capsicum*/*Curcuma longa* oleoresin mix	4 mg/kg	Increased body weight after CP challenge.	Reduced NE-related gross intestinal lesion score. Reduced inflammatory response against NE.	—	[48]

Citral (mix of *cis/trans*-isomers, 95% purity)	250–650 *μ*g/g	—	Reduced NE-related gross intestinal lesion score.	*In vitro* antibacterial activity against CP. Decreased CP counts in ileum and ceca. No effects in lactic acid bacteria.	[49]

Commercial EOs product (25% thymol and 25% carvacrol)	60, 120, or 240 mg/kg	No influence in growth performance.	Reduced NE-related gross intestinal lesion score.	*In vitro* antibacterial activity against CP.	[50]

Commercial herbal mix (thyme and star anise 17.0% and 17.0%)	250 mg/kg	Improved FCR. Improved digestibility of dry matter.	Reduced NE-related gross intestinal lesion score.	Decreased CP counts in large and small intestines.	[51]

EOs from *A. millefolium*	1 g/kg	Decreased BWG. Decreased FI. Improved FCR.	—	—	[52]

EOs from* O. majorana*	1 g/kg	Improved BWG and FI under suboptimal conditions for growth.	—	—	[52]

EOs from *O. vulgare* subsp. *hirtum*	1 g/kg	Decreased BWG. Decreased FI.	—	—	[52]

EOs from *R. officinalis*	1 g/kg	Decreased BWG. Decreased FI.	—	—	[52]

EOs from *T. vulgaris*	1 g/kg	Improved BWG under suboptimal conditions for growth.	—	—	[52]

EOs mix (thymol, cinnamaldehyde, and essential oil of eucalyptus)	150 g/ton	—	Reduced NE-related gross intestinal lesion score.	*In vitro* antibacterial activity against CP.	[53]

Marjoram (*O. majorana*)	10 g/kg	Improved BWG and feed conversion rate under suboptimal conditions for growth.	—	—	[52]

Oregano (*O. vulgare* subsp. *hirtum*)	10 g/kg	Decreased BWG. Decreased FI. Improved FCR.	—	—	[52]

Protected blend of EOs (ginger oil and carvacrol 1%)	1.5 g/kg	Improved BWG.	Reduced NE-related gross and histopathological intestinal lesion score. Increase in intestinal villus lengths and V : C ratio.	—	[54]

Rosemary (*R. officinalis*)	10 g/kg	—	—	Decreased CP counts in ceca and feces. No effect in lactic acid bacteria.	[52]

Thyme (*T. vulgaris*)	10 g/kg	Reduced BWG and FI under suboptimal conditions for growth.	—	Decreased CP counts in ceca. No effect in lactic acid bacteria.	[52]

Yarrow (*A. millefolium* var. *alba*)	10 g/kg	Improved BWG and feed conversion rate under suboptimal conditions for growth.	—	Decreased CP counts in ceca and feces. No effect in lactic acid bacteria.	[52]

CP: *C. perfringens*; BWG: body weight gain; FI: feed intake; FCR: food conversion rate; NE: necrotic enteritis.
